# Crack-Based Sensor with Microstructures for Strain and Pressure Sensing

**DOI:** 10.3390/s23125545

**Published:** 2023-06-13

**Authors:** Nakung Kim, Daegeun Yun, Injoo Hwang, Gibaek Yoon, Seong Min Kang, Yong Whan Choi

**Affiliations:** 1 Division of Mechanical Convergence Engineering, College of MICT Convergence Engineering, Silla University, Busan 46958, Republic of Korea; hjks@sillain.ac.kr (N.K.); ydg452@naver.com (D.Y.); hwanginjoo@silla.ac.kr (I.H.); s-201766049@sillain.ac.kr (G.Y.); 2 Department of Mechanical Engineering, Chungnam National University, Daejeon 34134, Republic of Korea

**Keywords:** bio-inspired, crack-based sensor, micropillar array, strain and pressure sensing, thin-film sensor

## Abstract

Recent extensive research on flexible electronics has led to the development of various flexible sensors. In particular, sensors inspired by the slit organs of a spider, which utilize cracks in a metal film to measure strain, have garnered considerable interest. This method exhibited significantly high sensitivity, repeatability, and durability in measuring strain. In this study, a thin-film crack sensor was developed using a microstructure. The results exhibited its ability to simultaneously measure the tensile force and pressure in a thin film, further expanding its applications. Furthermore, the strain and pressure characteristics of the sensor were measured and analyzed using an FEM simulation. The proposed method is expected to contribute to the future development of wearable sensors and artificial electronic skin research.

## 1. Introduction

Extensive research has been undertaken on small and flexible electronic devices [1,2,3], leading to their increased use in personal health management. In particular, research has been conducted on medical devices that measure the health status [4,5] of the elderly and detect diseases in advance. This has led to the development of sensors to accurately measure health status. These sensors can measure the strain or pressure levels and obtain information on muscle movement [6], heart rate [7], and blood vessel movement [8]. In addition, studies have been conducted to measure various changes and address the limitations of existing sensors, such as the detection of volatile organic compounds [9], the diagnosis of mechanical systems [10,11], wireless sensing [12], tactile perception [13], and terahertz biosensing [14]. There are various methods for fabricating these sensors, which can be classified into four main principles: using resistance changes [15,16,17,18], piezoelectric materials [19,20,21,22,23], capacitance changes [24,25,26], and the triboelectric principle [27,28,29,30].

Capacitance sensors operate on the principle that capacitance changes in proportion to the change in the distance between the two electrodes. A study was conducted on the production of graphene electrodes on both sides of poly dimethyl siloxane (PDMS) to demonstrate high sensitivity (3.19 kPa^−1^) and fast response time (30 ms) [27].

The method of using piezoelectric materials exploits the phenomenon where a potential difference is generated between both ends of the material when it undergoes a length change. A study was conducted on producing PbTi_3_ nanowires on graphene electrodes [21] to demonstrate high sensitivity (9.4 × 10^−3^ kPa^−1^) and fast response time (5–7 ms). However, piezoelectric materials are highly sensitive to temperature, and their sensitivity can change over time; therefore, additional research is needed.

Triboelectric sensors generate charges after friction or contact between two materials that become polarized when separated from each other. Electrons move to resolve the imbalance and generate electrical signals. Previous research has primarily focused on a form known as the triboelectric nanogenerator (TENG), which can be used as a self-powered sensor because it does not require an external power supply. Research on TENG using PDMS [30] has revealed its characteristics of being ultrathin (0.56 mm), ultralight (0.55 g), and flexible (a bending degree of 180°). As a result, the ultrathin sensor can be easily applied to human skin, and the movement of each joint, such as the fingers and wrists, can be measured.

When the sensor received an external force, the contact area of the conductive part of the sensor changed. This caused a change in the overall resistance, which was classified as a resistance sensor. Methods of utilizing resistance changes can be classified into strain and piezoresistive types.

Piezoresistive strain sensors based on laser-scribed graphene have also been reported. A strain sensor with a high gauge factor was fabricated using reduced graphene oxide (rGO) on PDMS. The calculated GFs for stretching, bending, and torsion were 12.1 and 3.5, respectively, and the strain range was 0–140% [18].

Another sensor that uses resistance changes and has received considerable attention is thecrack-based sensor. Spider legs have a slit organ that amplifies external stimuli. Crack-based sensors (CBSs) have been developed based on the structure of the slit organ [31]. A CBS consists of a polymer–metal bilayer and it easily generates cracks owing to differences in the elastic modulus between the two materials. The generated crack exhibits characteristics similar to those of the slit organs in spider legs and it amplifies the external force at the crack tip.

A crack-closed Pt film exhibited low resistance values when the crack was completely closed. However, when tensile force was applied, the crack lips opened, the electrical pathway decreased rapidly, and the resistance increased. Consequently, a high gauge factor (2000), short response time, and high durability were observed. Further research on CBSs can be applied to various application areas by changing the shape of the crack and the structure of the sensor. Previous research has been conducted on crack shapes with a focus on crack depth and density. Research has also been conducted on the crack depth by adjusting the pre-stretching force. It was fabricated by depositing 20 nm-thick Pt on a PUA substrate, and the depth of the crack varied depending on the degree of bending before testing [32]. As the depth of the crack increased, the amplification effect of the external stimuli increased; thus, deeper cracks exhibited higher sensitivity (GF 16,000 under 2% strain). An attempt was made to adjust the density of cracks using carbon nanotubes as the conducting layers [33]. By adjusting the number of layers, it was possible to control the density of the cracks. It was observed that as the number of layers decreased, the density of the cracks decreased and their length increased. Consequently, the sensitivity increased as the crack density decreased; however, the measurement range decreased.

Basically, a CBS is a form of strain sensor. Through the deformation of the structure of the sensor, the CBS can measure various parameters such as pressure [34,35,36,37,38,39], gas or chemical concentrations [40,41,42], temperature [43,44], and magnetic fields [45]. Various approaches have been used to measure pressure. A thin-film-type crack sensor was applied to the PDMS block with holes [34]. In addition, research has utilized the phenomenon in which cracks widen when pressure is applied by forming a Cu metal film network wire on PDMS [35]. The method of applying polyaniline nanohair (PANIH) was used to create a coat on a sponge between two GO layers [36]. In this case, when the pressure was low, the GO crack widened and the conduction between the two GO layers increased, whereas when a large pressure was applied, the PANIH applied to the sponge structure was pressed and the electrical pathway increased rapidly.

When pressure was applied to the CBS, the widening of the crack gap was not affected. Therefore, a method that converts the applied pressure into tensile stress through a structure such as a diaphragm on a hole or an anisotropic Poisson’s ratio substrate is mainly used. However, the pressure sensors manufactured using this method are thicker and have the disadvantage of low flexibility. As research on flexible electronics has progressed, flexible sensors with a thin form that does not cause significant strain on the surface when bent at a certain curvature have also been actively developed. Therefore, CBSs with high sensitivity and repeatability need to be developed as thin-film pressure sensors in thin-film form for flexibility.

In this study, these microstructures were applied to a CBS on a thin film. The microstructure was inspired by the hairy structures on the feet of gecko lizards, and micropillar arrays were applied on the opposite side of the CBS. Consequently, when pressure was applied to the micropillars, an imbalance in stress occurred between the pillars and pattern gaps. Owing to this imbalance, local strain changes occurred in the crack sensor when pressure was applied to the entire sensor, and the pressure level was detected. Fabrication of a thin film pressure sensor was possible by applying a microstructure to the thin film crack sensor (thickness 65 μm), and it was confirmed that the pressure sensitivity did not change even after 50 bending tests of 0.3 mm in radius. The change in resistance when strain was applied was analyzed, and the characteristics according to repetitive changes were studied. In addition, the sensitivity of the sensor when pressure was applied and the change in resistance with repetitive pressure were analyzed. The change in the pressure sensitivity was measured by changing the micropillar array, and an FEM simulation analysis was conducted to determine the cause of the pressure-sensing mechanism. Finally, it was demonstrated that the sensor could be applied to motion sensing applicationby applying it to the skin on the back of a hand to detect stretching and pressing.

## 2. Materials and Methods

### 2.1. Fabrication of a Strain and Pressure-Sensing CBS

**Figure 1:**
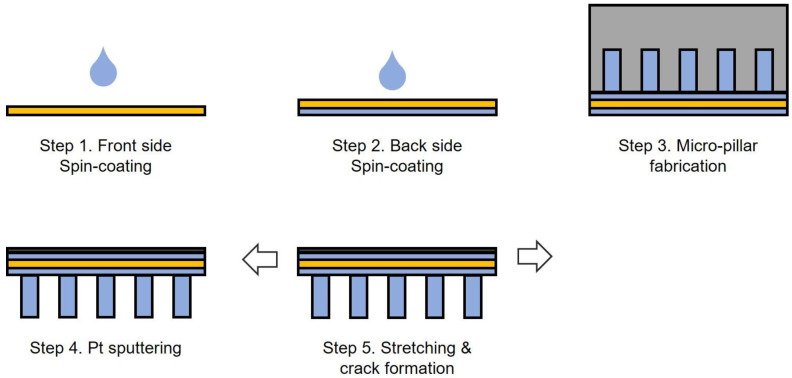
Fabrication process of the CBS with a micropillar array.

The Schematic illustrations of fabrication method is shown in Figure 1. A silicone rubber film of ~10 μm thickness was formed through spin-coating a solution of silicone rubber (KE-441, shinetsu) and tetrahydrofuran (Sigma–Aldrich, St. Louis, MO, USA) in a ratio of 1:8 (*w*/*w*) on a flexible polyethylene terephthalate (25 μm) film. The spin-coated film was cured in a dry oven at 70 °C for 3 h. Subsequently, a silicone rubber film was fabricated on the opposite side using the same method. The micropillar array was patterned using PDMS molds, which were prepared by replica molding from silicon masters fabricated through conventional photolithography. A mixture of base and curing agents (10:1, *w*/*w*, Sylgard 184, Midland, TX, USA) was poured onto the patterned silicon masters and cured in an oven at 70 °C for 1 h.

The surface of the cured PDMS mold was treated with trichlorosilane (1H,1H,2H,2H-perfluorooctyl; Sigma–Aldrich) in a vacuum chamber for 30 min. A micropillar array was obtained on the PET film via imprint lithography using a PDMS mold. Oxygen plasma was used for adhesion between the silicone rubber film and the PDMS.

On the opposite side, Pt was deposited by sputtering to an approximately 50 nm thickness. The deposited platinum thin film created a condition where cracks easily occur owing to the difference in the elastic modulus with the polymer film below. Therefore, when a tensile force is applied to the film, a crack is generated at the interface between the platinum thin film and polymer film, which subsequently propagates. When the film is repeatedly stretched under 2.5% strain, the cracks no longer propagate when the condition in which the cracks occur is saturated.

### 2.2. Electrical Characterization of a Strain- and Pressure-Sensing Sensor

The fabricated sensor was cut into dimensions of 5 mm × 20 mm and wired using conducting epoxy (CW2400, Circuitworks, Flemington, NJ, USA) for measurement purposes. Subsequently, the sensor was connected to a digital multimeter system (PXI-4071, National Instrument Inc., Austin, TX, USA) and fixed in the ultimate testing machine (UTM; 3342 Machine, Instron Co., Norwood, MA, USA) to measure the response to strain. The changes in resistance were recorded: the initial resistance of the sensor was 1 kΩ and the measurement range was set at 10 kΩ as it varied up to approximately 9 kΩ under 2% strain. A constant current of 1.0 × 10^−4^ A was applied for the measurement, and the voltage changes were measured and converted to resistance for recording. The response to pressure was evaluated by attaching the sensor to a testing bed and applying pressure to a 3 mm × 3 mm sample. The resistance changes were continuously measured by connecting the sensor to a digital multimeter. The applied pressure was calculated by dividing the force measured using the load cell connected to the sample by its area.

## 3. Results

### 3.1. Crack-Based Strain and Pressure Sensor

**Figure 2:**
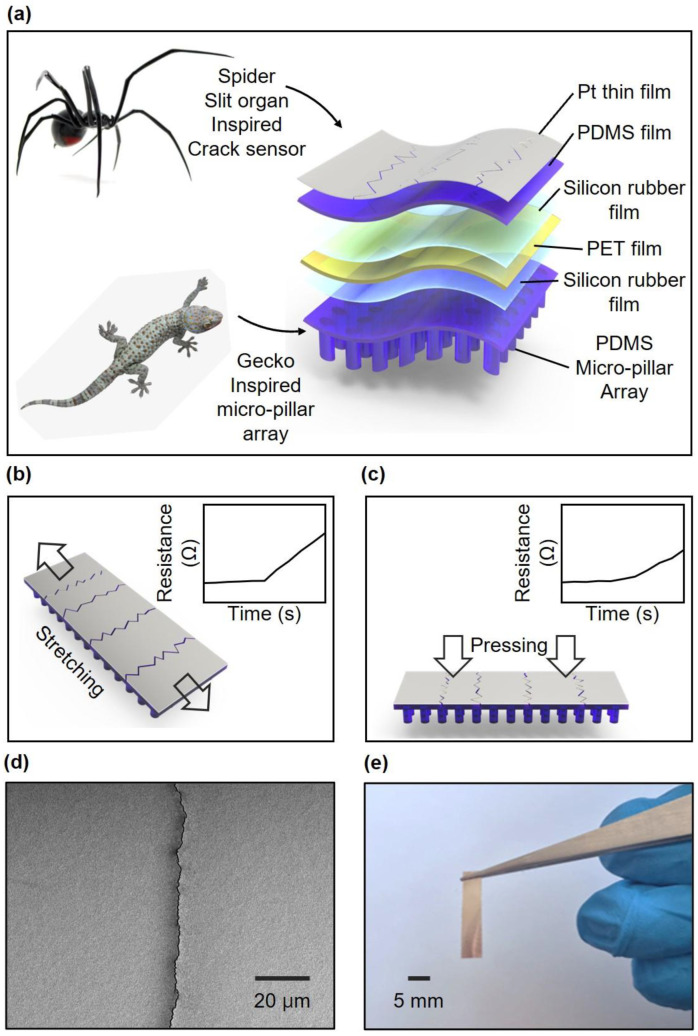
Illustrations and images of crack-based strain and pressure sensors. (**a**) Schematic of the slit organ of the spider- and gecko lizard-inspired structure. (**b**,**c**) Schematic of sensing strain and pressure and resistance changes (inset). (**d**) SEM image of cracked Pt/silicon rubber layer. (**e**) Photo image of the fabricated CBS.

Crack-based strain and pressure sensors can be divided into two types based on the middle film. In this study, the upper part was a strain sensor using a crack inspired by the slit organ of the spider, and the lower part was a micropillar array inspired by the hairy structure of the foot of the gecko lizard (Figure 2a). In previous research, a sensor inspired by the slit organ of the spider was made up of a double layer of polymer and a metal film with generated cracks. This crack structure amplifies the tensile force applied to the sensor and widens the gap between the crack lips. When the gap between the cracks widened, the number of electrical pathways in the metal film decreased, which was reflected in the geometrically exponential change in the platinum film resistance. The micropillar array at the bottom allows thin-film sensors to detect the pressure. This structure distributes pressure only across the micropillar portion and concentrates the stress on a portion of the upper film. Consequently, the pressure applied to the entire sensor exerts a slight strain on the film, increasing its resistance.

The effect of the micropillar structure was analyzed using FEM simulations. The schematic illustrated in Figure 2b shows that the sensor can measure tensile force. When tension was applied perpendicular to the direction in which cracks were generated, the resistance of the sensor increased, as shown in the graph inset of Figure 2b. The schematic illustrated in Figure 2c shows that the force was applied perpendicularly to the surface of the film-type sensor. In this case, although tension was not directly applied to the cracks, the resistance increased owing to the micropillar structure, as shown in Figure 2c (inset).

The crack structure allowed the sensors to measure the tensile force and pressure, and the SEM images taken at high magnification are shown in Figure 2d. The shape of the crack is illustrated, and it was observed that there was no difference in the resistance before and after the crack occurred when no force was applied. Figure 2e shows a photo image of the fabricated sensor, revealing its flexible nature and ability to be resized according to specific requirements. The sensor has dimensions of 5 mm × 20 mm, allowing for easy customization and adaptation to different applications.

### 3.2. AFM Analysis of Morphology or Crack-Based Strain and Pressure Sensors

**Figure 3:**
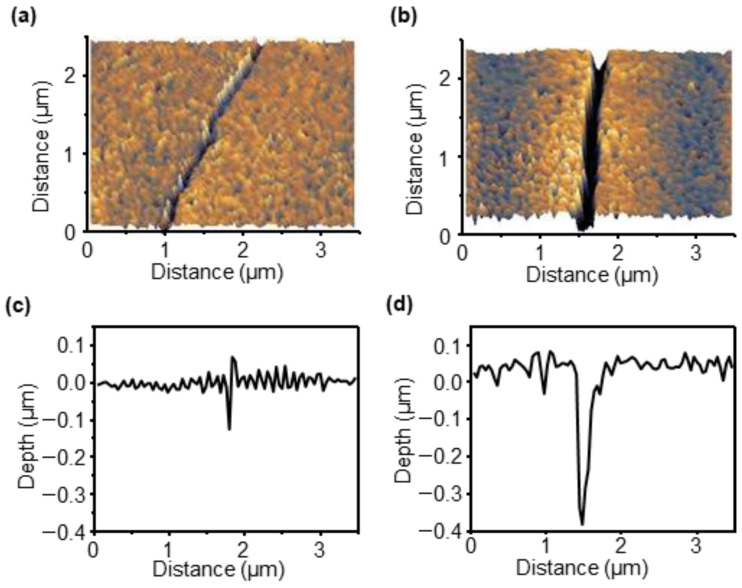
AFM data of the crack-based strain and pressure sensor. (**a**,**b**) Three-dimensional (3D) AFM image of the sample before stretching (**a**) and after stretching (**b**). (**c**,**d**) Line profile data of the sample before stretching (**c**) and after stretching (**d**).

The sensitivity of the sensor is significantly influenced by the morphology and depth of the crack. To observe the phenomenon of the crack gap widening and the increase in resistance as the sample was stretched, the crack shapes before and after stretching were compared using AFM measurements. The resistance before and after the initial crack in the sample remained unchanged, indicating that the crack remained fully closed when no strain was applied. This can be observed in the 3D image illustrated in Figure 3a and the line profile data presented in Figure 3c. As strain was applied, the crack gap gradually widened, and the morphology of the crack under 2% strain is shown in Figure 3b. The line profile data illustrated in Figure 3d show that, under 2% strain, a 194 nm wide and 381 nm deep crack appeared.

### 3.3. Characterization of a CBS for Strain Sensing

**Figure 4:**
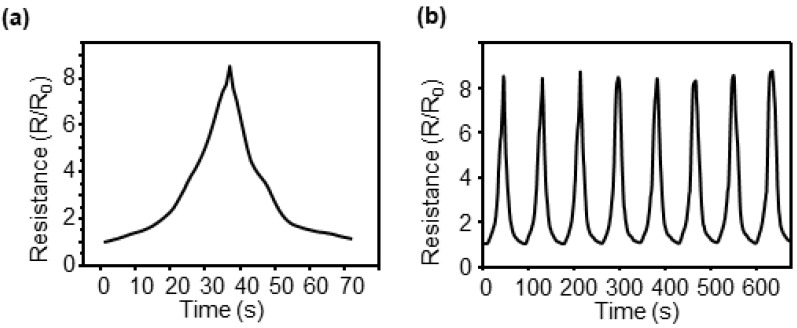
Resistance variation with strain of the CBS. (**a**) Loading/unloading behavior under 2% strain. (**b**) The normalized resistance change measured at 8 cyclic tests under 0–2% strain.

A UTM (3342 UTM, Instron Co., Norwood, MA, USA) was used to analyze the characteristics of the CBS for strain sensing. A constant strain was applied to the end of the sensor using the UTM, an electrical wire was connected to the sensor, and the resistance was measured by connecting the multimeter spontaneously. The resistance change measured using a multimeter during the analysis of the crack-based sensor characteristics for strain sensing is shown in Figure 4a. A resistance change was observed when the sensor was stretched by 2% and returned to its original position.

The resistance before the tension begins is known as R_0_, and the resistance change when 2% tension is applied to the sensor increases by 8.48 times compared with that of R_0_. The sensitivity of the strain sensor was determined using the gauge factor (R/R_0_/ε, ε = strain), and the calculated gauge factor was 424, which is significantly higher than that of the general strain sensor. The repeatability of the sensor was evaluated by determining the resistance change when 2% tension was applied eight times (Figure 4b) to analyze the characteristics of the sensor. During the iterations, the largest deviation in the gauge factor was 2.7% greater than the mean value, indicating that the results were uniform.

**Figure 5:**
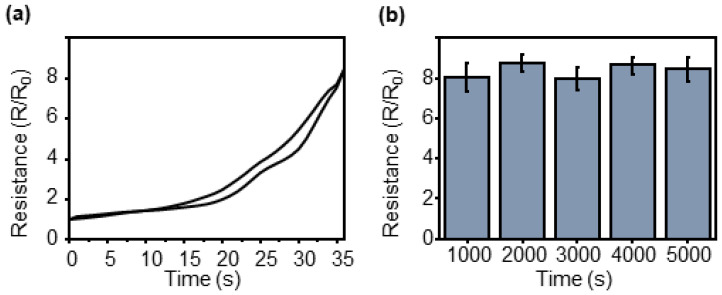
Repeatability test of the CBS. (**a**) Overlapped loading/unloading graph for hysteresis measure. (**b**) Marathon test of 5000 cycles.

Additional repeatability tests were conducted on the sensors. A graph showing the resistance change when tension was applied to the sensor and when the tension was removed during the measurement of hysteresis, which was determined as 9.67%, is illustrated in Figure 5a. A marathon test was conducted to evaluate the durability of the sensors over several repetitions, where the sensors were stretched 5000 times while recording the resistance change continuously (Figure 5b). The maximum change in the gauge factor during the test was 4.6% times compared to the average value.

### 3.4. Characterization of the CBS for Pressure Sensing

**Figure 6:**
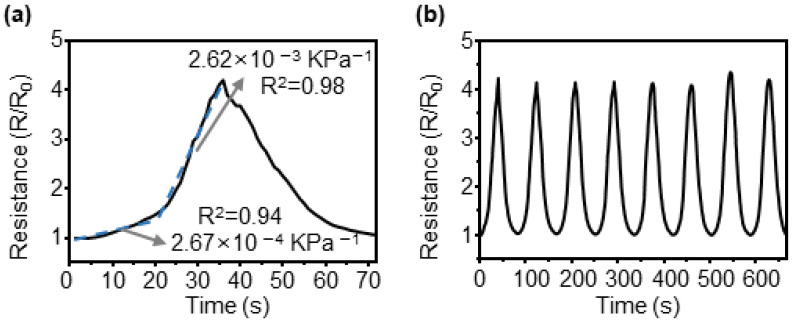
Resistance variation with pressure of the CBS. (**a**) Loading/unloading behavior under 2.18 MPa pressure. The dotted lines indicate the linear approximation. (**b**) The normalized resistance change measured at 8 cyclic tests under varying pressure.

To analyze the pressure-sensing characteristics of the CBS, tests were conducted using a custom-built machine with a load cell, and the reactive force generated by the sample was measured while precisely moving the input distance. The measured force was converted into pressure by dividing it by the applied area. The area subjected to pressure was 3 mm × 3 mm in size, and the maximum force measured by the load cell was 2 kg. When the applied force was converted into pressure, it resulted in a value of 2.18 MPa. Figure 6a shows the change in resistance over time when pressure was applied to the sample and subsequently removed. The sensitivity of the pressure sensor can be expressed as a change in the sensor value according to the applied pressure. For crack sensors, the resistance value changed from the initial resistance value before testing and was calculated as a sensor value. The resistance change of the sensor did not appear to be linear; however, it increased gradually in magnitude. In particular, at a pressure of approximately 1.2 MPa, the slope changes significantly, and sensitivity to pressure can be expressed in two parts based on this inflection point. When approximated linearly at a pressure range of 0–1.2 MPa, the slope was 2.67 × 10^−4^ kPa^−1^ and R^2^ was 0.94. The sensitivity at a pressure range of 1.2–2.18 MPa was 2.62 × 10^−3^ kPa^−1^ and R^2^ was 0.98. Figure 6b shows the results of measuring the resistance changes during eight repetitions to test the pressure detection repeatability of the sensor. The maximum change compared with the average value of the gauge factor during repeated tests was determined as 4%, which is significantly small.

**Figure 7:**
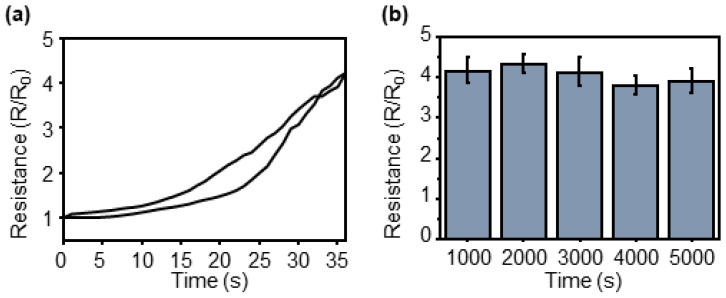
Repeatability test of the CBS for pressure sensing. (**a**) Overlapped loading/unloading graph for hysteresis measure. (**b**) Marathon test of 5000 cycles.

Additional experiments on the repeatability of pressure detection by the sensors are shown in Figure 7. Figure 7a shows the hysteresis obtained by overlapping graphs of the resistance changes when pressure is applied and removed. The hysteresis was determined to be 16.9%. It exhibited a larger value than that of the reaction to tensile stress. When pressure was applied to the sensor, the force applied to the micropillar structure was transmitted to the film by the crack structure. At this time, the micropillar structure received force, deformation occurred, and it was established that significant hysteresis occurred owing to this effect. Accurate analysis requires additional research through FEM simulations and experiments. To test durability under repeated pressures, a marathon test was conducted, and the results are shown in Figure 7b. Pressure was applied to and removed from the sensor for 5000 cycles, and the maximum change from the average value of the gauge factor calculated every 1000 cycles was 6.4%, indicating that the micropillar array was not destroyed even under repetitive pressures.

### 3.5. Sensitivity Comparison Owing to Micropillar Pattern Changes

**Figure 8:**
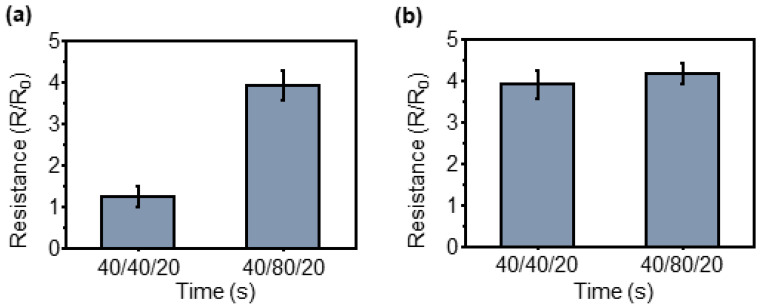
Sensitivity comparison according to pattern arrangement change. When the same pattern size/height and the spacing are different (**a**), the same pattern size/spacing and the height are different (**b**).

Because the sensor can detect the pressure applied by the micropillar array, the sensitivity also changes when the array changes its pattern. Figure 8 compares the sensitivity according to three pattern changes: the diameter of the circular pattern, the distance between patterns, and the height of the pattern.

Similarly, changes in resistance were observed when applying and removing a pressure of 2.18 MPa to the two differently patterned sensors. The results exhibited 2.95 times higher resistance change than when the pattern interval was 80 μm in comparison to when it was 40 μm. The graph illustrated in Figure 8b shows the sensitivity change according to the pattern height for the same pattern diameter and interval between patterns. Although the pattern with a height of 40 μm exhibited a 1.08-times higher resistance change than that of 80 μm, it was difficult to consider it as a significant difference. Therefore, it is evident that the sensitivity of the pressure sensor is significantly affected by the interval between patterns.

### 3.6. Thickness Comparison of the Sensor and Bending Test

**Figure 9:**
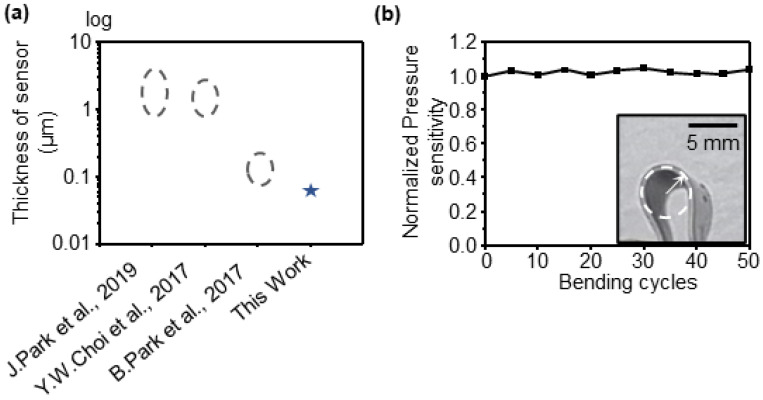
(**a**) Comparison of the thickness of sensors between the results obtained in this study and previous studies. The circle represents the thickness range of the sensor developed by the previous study, and the star represents the thickness of the sensor developed in this study. (**b**) Normalized pressure sensitivity as a function of bending cycles of 0.3 mm in radius was applied.

To confirm the advantages of the fabricated sensor, a comparison was made with those reported in previous studies. The thickness of the sensor, which is an essential requirement for flexibility, was compared, as shown in Figure 9a. Previous studies used a CBS as a diaphragm and applied it to a structure [46,47] or used an anisotropic Poisson’s ratio substrate with a thickness of approximately several mm to 100 μm [48]. In this study, the thickness was approximately 65 μm, which is thinner compared to previous studies, and it has the advantage as a flexible sensor. A bending test was conducted to verify the flexibility of the sensor. The sensor underwent repeated bending under the condition of a minimum bending radius of 0.3 mm (Figure 9b inset), and the pressure sensitivity after bending was compared with that before bending (Figure 9b). Consequently, it was confirmed that the pressure sensitivity remained unchanged, even after 50 bending cycles.

### 3.7. FEM Simulation Analysis of the Effect of Micropillar Pattern Array Variation

**Figure 10:**
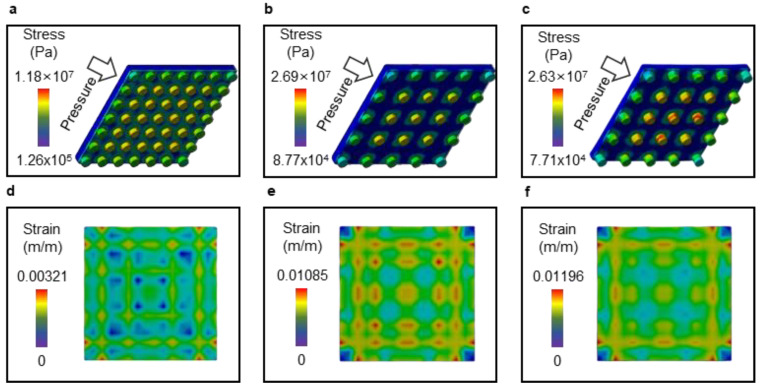
FEM simulation results for pattern variances. (**a**–**c**) Von Mises stress analysis for the whole sample. (**d**–**f**) Equivalent elastic strain analysis results of the top layer.

The cause of the pressure sensitivity difference according to the micropillar pattern change was analyzed using an FEM simulation. Static structural simulation analyses were performed using ANSYS, and pressure was applied to the upper surface of the micropillar while the bottom surface was fixed. To confirm the effect of cracks on the film, a silicone rubber/PET/silicone rubber/PDMS micropillar composite layer was modeled and verified. Because several deformations occurred in the PDMS micropillar structures, a large deflection was considered. This result is illustrated in Figure 10a–c. Analysis was performed for three patterns with pattern sizes/pattern intervals/pattern heights of 40/40/20 (Figure 10a,d), 40/80/20 (Figure 10b,e), and 40/80/40 (Figure 10c,f), respectively. The maximum stress and average values were higher for samples with wider pattern intervals. In addition, considering the stress distribution, the stress was concentrated around the micropillars, and this imbalance caused deformation in the upper film. To confirm the size of the deformation appearing on the upper film, the local equivalent elastic strain was analyzed, and the results are shown in Figure 10d–f. It can be confirmed that the strain results appear in a certain pattern owing to the micropillar structure, and their maximum values differ from each other.

It was observed that the maximum equivalent elastic strain was 3.38 times higher for patterns with an interval of 40/80/20 than for those with an interval of 40/40/20, which is consistent with the sensitivity difference measured earlier for pressure.

### 3.8. Applications

**Figure 11:**
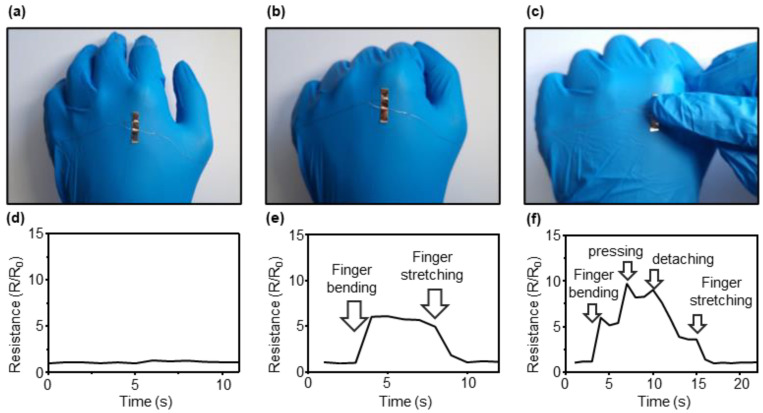
Response of the sensor to the movement of the finger and gentle pressing: (**a**–**c**) photo image and (**d**–**f**) resistance variations.

A flexible sensor can be used in various personal health management fields. Additionally, it is possible to measure and analyze movements and gestures. Tests were conducted to demonstrate that strain and pressure-sensing CBS can measure various stimuli attached to the skin, and the results are shown in Figure 11. When all the fingers were bent in the form of a fist from the open palm, the skin on the back of the hand was stretched. A sensor attached to the back of the hand measured this movement as a change in resistance. Photographs of the movements measured by the sensors attached to the back of the hand are shown in Figure 11a–c. A test was conducted to determine whether the changes could be measured by bending the fingers after attaching the sensors to the back of the hand. In addition, unlike general crack-based sensors that can measure the tensile strength as strain sensors, sensors with micropillar structures can measure the pressure and strain simultaneously. To demonstrate this, the resistance changes when pressure was applied by touching fingers while bending them were measured after attaching sensors to the back of the hand, as shown in Figure 11d–f.

## 4. Discussion

CBSs are an active field of research because of their high sensitivity, fast response time, and simple fabrication methods. CBSs are a type of strain sensor that measure length changes, and research on their modification to measure pressure, temperature, gas, and other factors has been active. One of the most actively researched areas is the development of pressure sensors that can be applied to detect touch in robots or acquire various bio-signals such as heart rate and blood vessel contraction. Although most of these applications require a flexible and thin fabrication method, they are not easily achievable because sufficient length changes in the direction of the applied force are required to detect the pressure. In this study, a micropillar structure was applied to a highly sensitive CBS. This sensor not only detected length changes but also measured the pressure perpendicular to the film surface. To analyze the characteristics of the sensor, we measured the changes in resistance under 0–2% strain conditions and analyzed the changes in response to repetitive stimuli. We also measured the resistance changes under constant pressure to observe the response of the sensor to pressure and its repeatability. The micropillar structure caused non-uniform stress on the film, resulting in local strain and an increase in resistance in the cracked areas. To verify this process, we analyzed three different patterns and compared the resistance changes in response to pressure. The results demonstrated that wider pattern intervals exhibited higher resistance changes, whereas changes in pattern height did not significantly affect the resistance. A finite element simulation was also performed to investigate the role of the micropillar array, and the results exhibited a trend similar to that of the resistance change results. The equivalent elastic strain analysis of the cracked films indicated that the pattern interval was the most important parameter for detecting pressure, whereas the pattern height changes were not as significant. Therefore, the hypothesis of the pressure-sensing mechanism is that the micropillar structure causes nonuniform stress in the film, and the pattern interval was observed to be the most critical parameter for the sensor array.

Additionally, to demonstrate the diverse applications of the sensor, we tested its ability to detect finger movements by attaching it to the skin of the hand. The sensor was able to measure skin stretching caused by finger movements and simultaneously detect the pressure applied by the hand.

## 5. Conclusions

We fabricated a thin-film strain/pressure sensor by incorporating micropillar structures into a CBS for strain measurements. The resulting sensor successfully measured the pressure in a thin-film form and exhibited exceptional flexibility. This outcome is significant because it demonstrates the effective application of the sensor on surfaces with low curvature, thereby expanding its potential applications. Furthermore, the existing limitations of CBSs can be overcome by applying various microstructures or shape modifications. Future research should focus on further reducing thickness, increasing sensitivity, and ensuring a consistent response to pressure even under bending conditions.

## Data Availability

The data presented in this study are available on request from the corresponding author.
